# Long-term performance of thermoacidophilic methanotrophic biotrickling filters

**DOI:** 10.3389/fbioe.2026.1954174

**Published:** 2026-10-29

**Authors:** Fletcher Gilbertson, Aaron T. Marshall, Carlo R. Carere, Peter A. Gostomski

**Affiliations:** Te Tari Pūhanga Tukanga Matū | Department of Chemical and Process Engineering, Te Whare Wānanga o Waitaha | University of Canterbury, Christchurch, New Zealand

**Keywords:** biofiltration, biotrickling filter, extremophile, methane, methanotroph, thermoacidophilic

## Abstract

This study investigated thermoacidophilic biotrickling filters (BTFs) for methane mitigation under acidic (pH 2.2–2.5) and thermophilic (45 °C) conditions. Four laboratory-scale BTF reactors were operated over ∼700 days under anaerobic, microaerobic, and aerobic regimes to evaluate the enrichment, activity, and stability of methane-oxidizing microbial communities. Following an initial establishment phase, biofilms developed successfully on pumice packing, with methane elimination capacities (ECs) reaching 41.9 ± 2.6 g m^-3^ h^-1^ under microaerobic conditions (1%–3% O_2_). Aerobic reactors achieved maximum ECs of 30.7 ± 2.4 g m^-3^ h^-1^, while performance declined at higher methane loading rates, likely due to oxygen limitation and accumulation of inhibitory intermediates such as formic acid. Microbial community analysis revealed persistent dominance (47%–99%) of the thermoacidophilic methanotroph *Methylacidiphilum*, despite their low initial abundance in the geothermal soil inocula. Reactor performance varied with time, driven by nutrient availability, biofilm accumulation and mass transfer limitations, including liquid channelling and bed flooding. Elevated CO_2_ recovery (>100%) and O_2_ consumption indicated a possible shift towards endogenous carbon metabolism and potential syntrophic interactions within the community. Additionally, iron dissolution within reactors supported the proliferation of iron-cycling acidophiles. Overall, this work demonstrates, for the first time, long-term operation of thermoacidophilic methane-removing BTFs and highlights their potential for treating hot, acidic gas streams such as landfill biogas and geothermal emissions.

## 1 Introduction

Methane (CH_4_) emissions have contributed ∼30% to the global temperature increases since the industrial revolution (IEA, 2025). Anthropogenic methane emissions come from a variety of sources (e.g., agriculture, fossil fuels, waste handling and biofuel burning) (Jackson et al., 2024). Methane emissions can be oxidized a variety of ways including combustion, photocatalysis and biological methods like biofiltration and biotrickling filters (BTFs) with the performance of different technologies concentration-dependent (Chen et al., 2016; Chi et al., 2012; de Almeida et al., 2025; Hickey and Allen, 2024; La et al., 2018).

Methane biofiltration uses the metabolic activity of aerobic methane-oxidising bacteria (methanotrophs), to convert methane into carbon dioxide (CO_2_), thereby decreasing the impact of the emissions (La et al., 2018). Similarly, biotrickling filters offer equivalent performance, while providing better control of operating parameters such as nutrients, temperature, and pH (Cáceres et al., 2017).

One of the challenges for methane biofiltration is effective mass transfer due to the low water solubility of methane which is ∼20 mg L^-1^ at 1 atm and ambient temperatures (Cesário et al., 1997; Estrada et al., 2014; Sander, 2023). Excessive water in the trickling layer can be a barrier to effective mass transfer (Ferdowsi et al., 2017). Various modifications to traditional biofilters and biotrickling filters have been implemented to alleviate this limitation including supplementing with surfactants and non-aqueous liquids (Kraakman et al., 2025; Lamprea Pineda et al., 2021; Lebrero et al., 2015) capillary reactors (Kraakman et al., 2023), aerosol nutrient delivery (Pryor et al., 2026) and fungal communities (Marycz et al., 2023).

Common methane contaminants, such as H_2_S, can be inhibitory to the mesophilic methanotrophs abundant in traditional methane BTFs (Cáceres et al., 2014; Pei et al., 2022). Thermoacidophilic methanotrophs in the genus *Methylacidiphilum* (phylum: Verrucomicrobiota) are naturally acclimated to sulphurous environments (e.g., H_2_S emitted from geothermal soils) (Carere et al., 2017; Mohammadi et al., 2017; Schmitz et al., 2021), are known to oxidize H_2_S (Kalyuzhnaya et al., 2013; Schmitz et al., 2022), so may provide an advantage in methane biofiltration applications. Thermophilic methane treatment from municipal landfill gases is another possible application, as extraction and treatment of hot gases laden with methane tend to be acidic due to cocurrent H_2_S oxidation (Krause et al., 2023; Nikiema et al., 2007). In an application such as this, treating hot and acidic landfill biogas using thermoacidophilic BTFs would prevent emission of methane into the atmosphere and possibly improve landfill processes.

Both thermophilic and acidophilic BTFs are reported. Acidophilic, H_2_S-removing BTFs have been frequently investigated for the treatment/upgrading of biogas (Bhattarai et al., 2018; Dupnock and Deshusses, 2020; Kim and Deshusses, 2005; Pol et al., 2007) and thermophilic methane removal at circumneutral pH has been summarised by Avila-Nuñez et al. (2026) at temperatures up to 60 °C (elimination capacity (EC) ranging from 38–75 g m^-3^ h^-1^). Regarding acidophilic methane removal, Syed et al. (2016) reported an EC of 30 g m^-3^ h^-1^ at pH 3.7 (ambient temperatures). Finally, the treatment of methane for the purpose of single cell protein production has been reported in a submerged bioreactor using *Methylacidiphilum* sp. RTK17.1 in coculture with an acidophilic microalgae (*Galdieria* sp. RTK37.1) at 45 °C and pH 2.5 (Cartin-Caballero et al., 2025). Despite these studies, the performance of thermoacidophilic methane-removing BTFs has never been reported. Therefore, the objective of this study was to enrich thermoacidophilic, methane-oxidizing communities in BTFs under microaerobic and aerobic conditions, and to characterize their metabolic activity and community composition for potential application in methane abatement technologies.

## 2 Materials and methods

### 2.1 Biotrickling filter reactor design and construction

The custom biotrickling filters (BTF) used in this study were based on prior studies (Liu et al., 2015; Wang et al., 2014). Briefly, the column BTF reactors were constructed from borosilicate glass with an inner diameter (ID) of 53.5 mm and a total length of 450 mm. Butyl rubber stoppers were fitted onto either end and secured using stainless steel compression plates secured via three threaded rods. Packing materials within each column were suspended by polytetrafluoroethylene (PTFE) plates perforated with ∼1 mm holes to allow liquid passage. Prior to using PTFE plates, stainless steel grates were used to support the packing material, however due to metal corrosion, the plates were replaced with inert PTFE. Stainless steel tubing (1/4″) threaded with M5 SMC nipple ports were bored through the butyl rubber stoppers on either end for gas and liquid flow (SMC, USA).

Two packing material types were tested: 2-mm glass beads and pumice rock (Daltons™ Coarse Pumice, New Zealand). Prior to use, the pumice rock was washed and screened to a consistent particle size (4–7 mm). Glass beads were used for the first 120 days of operation, however, due to a lack of biofilm attachment and poor reactor performance, pumice rock was subsequently used for the entirety of operation thereafter. The packing materials occupied ∼50% of the reactor volume (between 450 cm^3^and 495 cm^3^), including interstitial space between packing granules.

All gas streams were connected to the reactors via 4-mm ID fluoropolymer coated nylon tubing (SMC, IN, USA) to minimise gas permeability. Liquid streams were transported via Norprene™ or Pharma™ tubing, which have relatively low gas permeability rates and excellent chemical stability under acidic pH (Cole-Parmer, IL, USA). Liquid sumps were constructed from round bottom flasks, fitted with glass side ports for temperature and sensor probes, and contained the nutrient medium. Laboratory grade rubber stoppers (Cole-Parmer, IL, USA) sealed the top of the sumps and stainless-steel pipes or 4-mm glass tubes were bored through the stopper for transport of liquid or gas streams. The nutrient medium was circulated from the sumps to the top of the BTF reactors using a 4-channel Gilson MINIPULS three peristaltic pump (Gilson, WI, USA). Medium was distributed over the top of the BTF reactor bed using a four-port liquid nozzle connected to a threaded stainless-steel pipe. Additionally, Y−connectors with Luer-Lok™ sampling ports were placed inline of the Norprene™ liquid distribution lines to enable aseptic sampling.

Gas was supplied to the reactors in two different configurations. During the first 120 days of operation, pre-mixed certified calibration standard (CCS) 4% (±0.08%) v/v CH_4_ in N_2_ (BOC, NZ) was mixed with CCS grade 3% (±0.1%) CO_2_ (balance air) using Dwyer RMA-151 rotameters (Dwyer, USA). For the remainder of operation (∼1.5 years), gas was supplied from Ultra-High-Purity CH_4_ (Southern Gas Services, NZ), industrial grade CO_2_ and N_2_ (BOC, NZ) and compressed air mixed at appropriate flow rates using rotameters, along with Alicat™ and MKS mass flow controllers (MFCs) (Alicat, USA; MKS Instruments, USA). The N_2_ background gas for all reactors was supplied using rotameters, which were all connected to a cylinder and dual-stage regulator to minimize regulator outlet pressure variations. Before entering the reactors, combined gas streams were filtered via 50-mm Whatman™ 0.22-µm air filters (Whatman, UK) (Supplementary Figures S1 and S2).

All BTF reactors were housed within a custom-built incubator and maintained at 45 °C (±0.4 °C) using two fan heaters connected to a proportional integral differential controller (XMT7100, Shanghai, China) and K-type thermocouple (SPK603 Shanghai, China). Each reactor was serviced with online sensors monitoring inlet/outlet gas composition (CH_4_, CO_2_, and O_2_), temperature, humidity and pressure. A Honeywell™ Sensepoint XCD flammable gas sensor (USA) was used in the incubator to detect large methane leaks and shut-off gas flow to avoid creating an explosive environment in the incubator. Further details described in Gilbertson et al. (2025).

### 2.2 Thermoacidophilic BTF reactor operation

BTF reactors were operated with counter current flows of gas (upward) and liquid medium (downward, 25 mL min^−1^) with the conditions outlined in Table 1. V4 nutrient medium was prepared according to Dunfield et al. (2007) with the addition of 0.2 mM rare earth elements (Pol et al., 2014) and the exclusion of yeast extract to minimise heterotrophic growth. V4 medium was prepared in Milli-Q water (18.2 MΩ cm) and contained (per litre) 0.4 gNH_4_Cl, 0.05 g KH_2_PO_4_, 0.02 g MgSO_4_·7H_2_O, 0.01 g CaCl_2_·2H_2_O, 3 mL Fe(II) EDTA solution, 3 mL trace elements one solution, 1 mL trace elements two solution, 200 µL 1 mM Ce(SO_4_)_2_, and 200 µL 1 mM Ln(SO_4_)_2_. The medium was finally adjusted to pH2.5 using H_2_SO_4_. The FeEDTA solution contained (per litre) 1.54 g Fe(II)SO_4_·7H_2_O and 2.06 g Na_2_EDTA. Trace elements solution 1 (pH2.5) contained (per litre) 0.44 g ZnSO_4_·7H_2_O, 0.20 g CuSO_4_·5H_2_O, 0.19 g MnCl_2_·4H_2_O, 0.06 g Na_2_MoO_4_·2H_2_O, 0.10 g H_3_BO_3_ and 0.08 g CoCl_2_. Trace elements solution 2 (pH2.5) was modified from Wolin et al. (1963) and contained 1.5 g nitrilotriacetic acid, 0.2 g Fe(NH_4_)_2_(SO_4_)_2_·6H_2_O, 0.2 g Na_2_SeO_3_, 0.1 g CoCl_2_·6H_2_O, 0.1 g MnSO_4_·2H_2_O, 0.1 g Na_2_MoO_4_·2H_2_O, 0.1 g Na_2_WO_4_·2H_2_O, 0.1 g ZnSO_4_·7H_2_O, 0.04 g AlCl_3_·6H_2_O, 0.025 g NiCl_2_·6H_2_O, 0.01 g H_3_BO_3_ and 0.01 g CuSO_4_·5H_2_O.

**TABLE 1:** Experimental setup and operating parameters of thermoacidophilic methane BTFs using pumice rock as a packing material.

Parameter	Value
Temperature (°C)	45
pH	2.2–2.5
Inlet relative humidity (%)	>70
Inlet gauge pressure (kPa)	0.07–0.12
Liquid flow (mL min^-1^)	15–25
Gas/liquid flow	Counter-current
Empty bed volume (mL)	510
Bed height and internal diameter, h x d (cm)	22 × 5.5
Liquid sump volume (L)	0.8–1.2
NH_4_^+^ concentration (g L^-1^)	0.127

V4 medium in the reactor sumps was regularly replaced, at a frequency (∼1–2 weeks) depending on the available ammonium content. On two occasions, reactor sumps were operated as chemostats to monitor the impact of nitrogen limitation on reactor performance. During this time, V4 medium was continuously supplied to each reactor sump at 0.05–0.2 mL min^-1^ using a precision 4-channel IPC Ismatec™ peristaltic pump with 1.14 mm ID silicone tubing (Ismatec, Germany). Two scavenger Huiyu BT100J-1A peristaltic pumps (equipped with YZ15-13A roller heads) were used to remove effluent medium at > 0.2 mL min^-1^ (Beijing Fluid Equipment Co. Ltd, China) using 3.2-mm ID platinum-cured silicone tubing.

Handling of the reactors and microorganisms was performed using aseptic techniques, whenever practical. Prior to inoculation, each packed reactor column was sterilised by autoclaving (121 °C, 15 psi for 30 min) and then dried in an oven at 70 °C overnight. All rubber bungs with metal gas/liquid line connectors and Norprene™ peristaltic pump tubing was covered in aluminium foil and autoclaved under the same conditions. After the columns had cooled to room temperature, reactors were assembled in a bio-cabinet. Fully assembled sterile BTF reactors were confirmed gastight by using Snoop™ and measuring inlet/outlet gas flow rates with a Bubble-o-meter™ (Bubble-o-meter, USA). The difference between inlet/outlet gas flow rates for all reactors was <5%. To confirm no abiotic methane loss, methane-laden gas streams were passed through reactors in the absence of inoculum. Changes in inlet/outlet gas composition were <0.13% (v/v) for CH_4_, O_2_ and CO_2_.

All BTF reactor inlet gas streams were humidified and contained 1%–4% CH_4_ and 1%–3% CO_2_ in N_2_ with three reactors also receiving 2%–10% inlet O_2_ (all v/v). This range of CH_4_ ensured methane limitation was unlikely and CO_2_ was added to ensure sufficient CO_2_ for autotrophic growth as commonly reported in thermoacidophilic methanotrophy (Khadem et al., 2011). Reactor 1 (R1, ‘anaerobic’) was operated without air in the gas inlet and was found to contain <500 ppm O_2_ concentration, due to diffusion/leakage into the reactor, so was not maintained at strictly anaerobic conditions. Reactor 2 (R2) was operated under ‘microaerophilic’ conditions (1.5%–3% v/v O_2_), while Reactors three and 4 (R3, R4) were operated aerobically (8%–12% v/v O_2_) as these columns are served as biomass sources for mediator studies and the enrichments used this O_2_ concentration range (Gilbertson et al., 2025). R1 and R2 periodically received no oxygen during days 600–700 due to thionine addition (Gilbertson et al., 2025). To avoid CH_4_ limitation within the column, gas flow rates were periodically increased as biofilm developed (Supplementary Table S1). Total gas flow rates for all reactors varied between 10–80 mL min^-1^ with a <2% rate difference observed between inlet and outlet streams. Three-way SMC 12 V solenoid valves were used to divert gas flow through the sump intermittently to quantify sump methane removal (Supplementary Figure S1). Gas streams were selected and run through an array of sensors using an eight-port flow-through valve, dead ended on each exhaust port for this application (VICI Valco™, USA). To protect the gas sensors and valves from water damage due to condensation when reactor gas streams dropped from 45 °C to room temperature, all gas streams passed through a refrigerator set to 4 °C before entering the sensor chamber (Supplementary Figure S1).

Towards the end of operation (between days 650–700), a redox mediator dye (600 μM thionine acetate) was used to visualise liquid channelling in the filter bed and assess mass transfer limitations in the BTFs.

Liquid sump and filter bed methane loading rates (LR) were varied between 10.5 and 353.2 g m^-3^ h^-1^ for all reactors, while inlet O_2_ and CO_2_ concentrations were held constant (Supplementary Table S1). This process was automated and measured daily for 1 week. The results were averaged, and sample standard deviations calculated over a week for EC, CO_2_ production, and O_2_ removal measurements based on CH_4_, CO_2_ and O_2_ concentration measurements along with error propagation in the relevant rate calculations. Further details are provided in Gilbertson (2023).

### 2.3 Gas analysis, automation and software

Data collection and reactor control was performed using a Raspberry Pi™ three running Raspbian 9. USB serial protocols (RS-232) (Raspberry Pi Ltd., UK) were used to communicate between the SGX gas sensors, VICI Valco valve, Gilson peristaltic pump, and Alicat™ MFCs. Phidget devices were connected to the Raspberry Pi via a Phidget interface module (VINT) running a proprietary communication protocol. A custom Python program was designed to control the BTF reactors, continuously collect, and analyse data. For gas analysis (CH_4_, CO_2_, O_2_), gas sensors (SGX Sensortech, CH) were connected to proprietary printed circuit boards (PCBs), containing the software and hardware for controlling the infra-red (IR) and electrochemical gas sensors (EC). SGX Sensortech EK-2 and EK-3 PCBs controlled the IR hydrocarbon/CO_2_ and EC O_2_ sensors, respectively. All PCBs supported RS-232 communication and were designed to minimize electrical noise and compensate for gas temperature variation (Gilbertson, 2023).

### 2.4 Soil collection and BTF inoculation

Soil samples were collected from geothermally heated soils in Rotokawa, New Zealand and stored at 4 °C as described previously (Carere et al., 2017). For BTF reactor inoculation, soil samples (54 g) were suspended in 1 L of sterile V4 medium. After mixing, the soil slurry was adjusted to pH 2.5 using 10 M KOH and sieved to <1 mm particle diameter to remove large soil granules.

Each reactor was then inoculated with 800 mL of soil slurry, which was recirculated in the reactors for 30 days, until CH_4_ removal was detected and biofilm development was observed. Sump and biofilm samples were periodically examined using a Primo Star 3 phase light microscope (Zeiss, Germany) in phase contrast mode equipped with a HD300 digital microscope camera (YSC Technologies, USA).

### 2.5 Analytical techniques

To quantify biomass, cell dry weights (DCWs) were calibrated against optical density (OD) at 600 nm. Briefly, 50-mL samples were collected from BTF reactor sumps, centrifuged at 8000 rpm (4 °C) for 12 min and then suspended in fresh V4 medium. The resulting microbial suspension was then serially diluted in triplicate, the OD was measured spectrophotometrically at 600 nm, and aliquoted into pre-weighed Eppendorf™ tubes. Samples were then centrifuged at 10,000 rpm (4 °C) for 5 min, the supernatant discarded, and the resulting and pellets were then dried to constant weight at 80 °C. One unit of OD_600_ was found to be equivalent to 0.43 g L^−1^ cell dry weight (R^2^ = 0.98).

The pH was measured using an CP-105 waterproof pH-meter with a GPX-105 probe (Elmetron, PL) throughout experimentation.

Molecular Fe was measured using an Agilent™ 4210 Microwave Plasma Atomic Emission Spectrometer. Samples were acidified (pH < 2) using 10% HNO_3_ (v/v) and compared against an external standard containing fresh V4 medium with a total Fe concentration of 2 ppm. All samples were run in triplicate. The Fe concentration was quantified by measuring the sample emission intensity at 372 nm using a charge-coupled device detector. The sample uptake time was set to 70 s and pump speed to 15 rpm, with a sample nebulizer flow of 0.85 L min^-1^.

High performance liquid chromatography (HPLC) was performed to quantify organic metabolites using a Dionex Ultimate 3000 HPLC (Thermo Scientific, Germany) equipped with a RefractoMax 520™ and a UV-VIS light detectors. A 0.3-m Aminex™ HPX-87H column was used with 5 mM H_2_SO_4_ mobile phase at a flow rate of 0.5 mL min^-1^ (Bio-Rad Laboratories Inc., USA). Chromeleon 7.2 SR5 software was used for data acquisition. To identify metabolites, the retention time of observed peaks was compared against corresponding standards of common metabolites in methanotroph metabolism (e.g., acetate, methanol, ethanol and formate).

### 2.6 DNA sequencing and community analysis

Biofilm samples were periodically harvested from the BTF liquid sumps to assess the microbial community structures present. These samples were centrifuged at 8000 rpm (4 °C) for 12 min, snap frozen in liquid nitrogen and stored at −80 °C until required for DNA extraction. Total genomic DNA was extracted from the soil inoculum and BTF biofilm samples using a DNeasy UltraClean Microbial Kit (Qiagen, Germany) according to the manufacturer’s recommended protocol. The quality of the DNA extracts was then evaluated using a Nanodrop One spectrophotometer (Thermo Fisher Scientific, USA). Samples with a 260/280 ratio of ∼1.8 and 260/230 ratio of >1.8 were considered acceptable. Purified DNA extracts were then tested using the Qubit™ dsDNA High Sensitivity Quantification Assay Kit (Invitrogen™, Thermo Fisher Scientific, USA) and quantified using the Qubit™ 4 Fluorometer (Invitrogen™, Thermo Fisher Scientific, USA) before being stored at −80 °C until required for DNA sequencing.

DNA extracts (>10 ng μL^-1^) were sent to the Auckland Genomics (NZ) for 16S rRNA gene amplicon sequencing of the V3-V4 hypervariable region. Dual index PCR primers were used to flank the V3-V4 hypervariable region of 16S rRNA. The “Single Step PCR Library preparation method” was used (Kozich et al., 2013) to prepare sample libraries. The primer sequences used were 16Sf V3 (CCT​ACG​GGA​GGC​AGC​AG) and 16Sr V4 (GGACTACHVGGGTWTCTAAT) (Integrated DNA Technologies, USA). Sample libraries were pooled by equal molarity and quality control before being analysed using the MiSeq 2 × 250 base PE Nano run, version two chemistry (Illumina, USA). To prevent uneven representation of bases at each cycle with amplicon sequencing, an Illumina PhiX control library was loaded onto the Illumina Miseq run at 20% volume to even out the base composition and prevent base calling errors.

Processing of the sequence data was conducted by Environmental Science and Research (NZ) using a custom pipeline (Wigley et al., 2022). Briefly, sequence data was processed using BBduk (Bushnell, 2014) and trimmed at an error probability of 0.01 (Phred score of Q20) using the “dynamictrim” application from the SolexaQA++ software (Cox et al., 2010). Using the dada2 program (Callahan et al., 2016) on RStudio (R Core Team, 2023), microbial taxonomy was assigned to the sequence variants using Silva 138.1 database (Yilmaz et al., 2014). Alpha diversity metrics (Shannon entropy and Simpson diversity) and Bray–Curtis dissimilarity matrices were calculated using the *phyloseq* package (McMurdie and Holmes, 2013) in RStudio (R Core Team, 2023), with outputs exported and visualised in Prism version 10.1.2 (GraphPad Software, USA).

All biotrickling filter performance calculations (e.g., EC, LR, RE, CO_2_ Recovery) are outlined in Supplementary Equations S1-5.

## 3 Results and discussion

This study investigated how O_2_ supply, CH_4_ loading rate and NH_4_^+^ availability impacted the establishment and performance of thermoacidophilic (pH 2.2–2.5, 45 °C) methane-removing biotrickling filters over a 2-year period. Aside from the initial biofilm development phase, the methane removal efficiency (RE) for all reactors were maintained at < 100% to ensure CH_4_ was not limiting within the filter bed. For all reactors, CH_4_ removal from the liquid sumps was negligible.

### 3.1 Biofilm establishment and initial BTF performance

Prior to using pumice rock, 2-mm glass beads were trialled as the packing material in BTF reactors R1 and R2. During this initial 120-day period, no significant methane removal was detected, and no visible biofilm development or turbidity increase in the liquid sumps was observed. Although glass bead packing materials exhibit poor surface area to volume ratios and water holding capacities (López de León et al., 2019), they are commonly used in conventional BTF studies as they provide an inert and well-defined structure for model development (Lee and Heber, 2010). The reason why biofilm growth was inhibited on the glass beads was unclear, however, under acidic conditions (pH < 3) glass surfaces display positive zeta potentials and charge densities (Behrens and Grier, 2001; Bender, 1954). Bacterial cell outer membranes are generally negatively charged at pH 7, which thus facilitates attractive electrostatic interactions with glass beads and promotes biofilm development at circumneutral pH values (Wilson et al., 2001). In contrast, acidophiles, such as *Acidithiobacillus ferrooxidans*, can display neutral and increasingly positive surface charge at pH < 3 (Skvarla et al., 2002). *Acidithiobacillus* spp. were abundant (∼80%) in the geothermal soil samples used to inoculate the BTFs in this study, are ubiquitous in acidic environments and are often dominant in acidic BTF studies (Arespacochaga et al., 2014; Nagendranatha Reddy et al., 2019; Zhang et al., 2022). Considering this, it was possible that repulsive microbe-glass electrostatic interactions contributed to the lack of biofilm development during the initial enrichment period. Thus, glass beads were replaced with pumice rock, which also offered the benefit of increased surface roughness, and porosity.

After switching to pumice rock as a packing material, biofilm developed in reactors R2, R3 and R4 within 35 days. Correspondingly, BTF methane elimination capacities (ECs) also increased during this period and reached stable readings of 4.8 (±2.1), 24.1 (±1.2) and 23.3 (±1.1) g m^-3^ h^-1^, for R2, R3 and R4 respectively between days 30 and 55 (Figure 1) at a loading rate of 33.4 (±2.7) g m^-3^ h^-1^. During the same period of observed biofilm accumulation, O_2_ consumption and CO_2_ generation increased (Figure 1). On day 75, the methane loading rate for all reactors was decreased by ∼25% (Supplementary Table S1). This decrease in CH_4_ loading rate coincided with a decrease in EC in R3, likely reflecting the diminished driving force slowing mass transfer of CH_4_ into the biofilm (Cesário et al., 1997; Gómez-Cuervo et al., 2016). However, the EC in R1, R2 and R4 along with O_2_ consumption and CO_2_ generation in all the reactors was not statistically different after the loading rate decrease (p > 0.05). Nevertheless, continued biofilm accumulation in the BTF beds led to increased pressure drop and bed flooding, necessitating occasional inversion of the beds to dislodge excess biofilm (Supplementary Figures S3-7). Contrasting non-extreme methanotrophic BTFs (Ferdowsi et al., 2017; López et al., 2018; Pryor et al., 2026), where biofilm growth and associated increased pressure drop is normally minimal despite an excess nutrient supply, within the thermoacidophilic BTFs, biofilm growth continued unabated throughout their operation.

**FIGURE 1:**
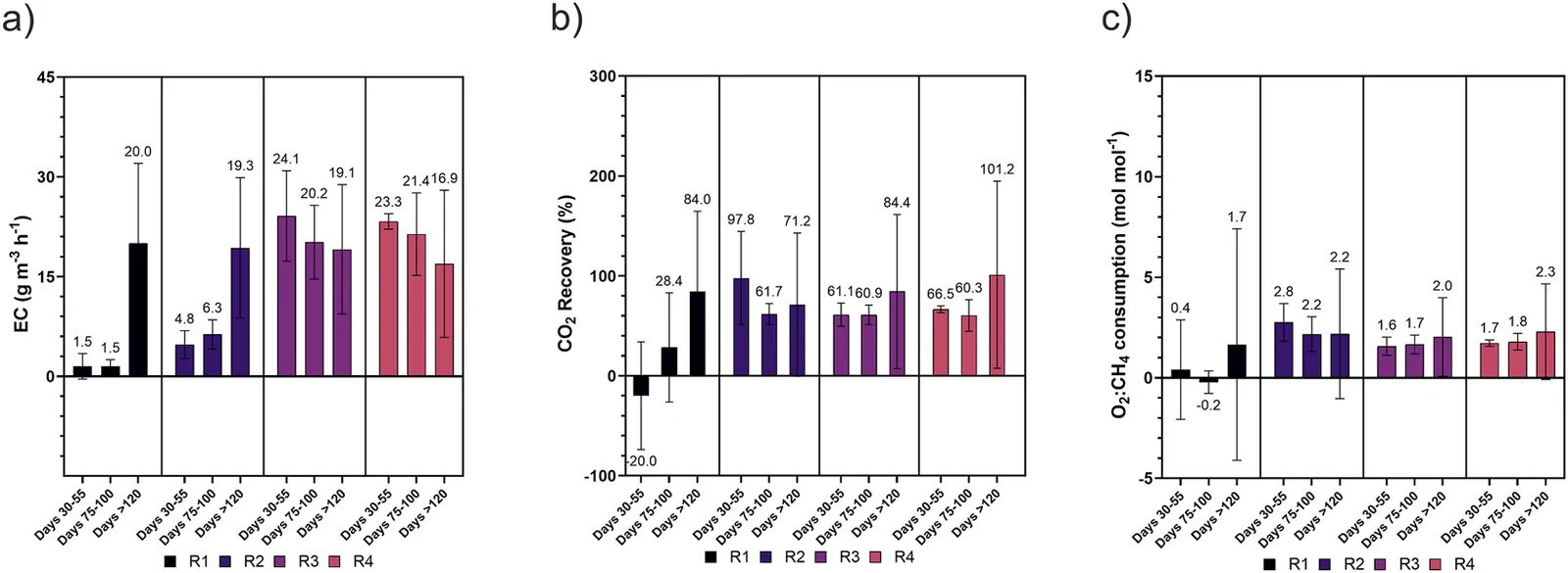
The **(a)** methane elimination capacity (EC), **(b)** CO_2_ recovery (%), **(c)** and O_2_:CH_4_ consumption ratio in BTF reactors R1, R2, R3 and R4 during three different operational periods (Days 30–55, Days 75–100, Days >120) with pumice as a rock packing material. During days 30–55, a methane loading rate of 33.4 (±2.7) g m^-3^ h^-1^ was used. While from days 75–100, the methane loading rate was 23.8 (±3.4) g m^-3^ h^-1^. Loading rates >120 days ranged between 10.5 (±0.53) and 353.2 (±1.61) g m^-3^ h^-1^ (Supplementary Table S1). Average values are shown with error bars representing one standard deviation (Days 30–55, n > 50; Days 75–120, n > 50; Days >120, n > 1000).

Throughout the initial establishment period, Reactor one was operated ‘anaerobically’ with no air supply. O_2_ was detectable at < 500 ppm O_2_, due to diffusion/leakage into the reactor. These conditions were maintained for the first 300 days (Figure 2). Methane removal during ‘anaerobic’ operation was minimal (1.17 ± 0.48 g m^-3^ h^-1^) and attributed to the minimal O_2_ present. Anaerobic methanotrophs are slow growing (μ ≈ 0.001 h^-1^), compared to their faster growing aerobic counterparts (μ ≈ 0.1–0.4 h^-1^) (Girguis et al., 2005). Thus, it seems reasonable the minimal O_2_ promoted the growth of aerobic methanotrophs to the detriment of anaerobic biofilm development (Cassarini et al., 2019; Girguis et al., 2005; Hou et al., 1979; Strong et al., 2016). Hence, on day 302, R1 was switched to ‘microaerobic’ operation and operated in parallel to R2 (1%–3% inlet O_2_, v/v) for the remaining duration of the study. In the first 35 days of microaerobic operation, the R1 EC rapidly increased 9-fold to 11.1 (±0.7) g m^-3^ h^-1^ (Figure 2). On day 320, the RE reached 96% and to avoid CH_4_ limitation, the LR was increased to 52.4 (±1.0) g m^-3^ h^-1^. This resulted in a stable EC of 42.0 (±2.4) g m^-3^ h^-1^ for the next 33 days. This was the largest sustained and stable EC experienced among all reactors during the study.

**FIGURE 2:**
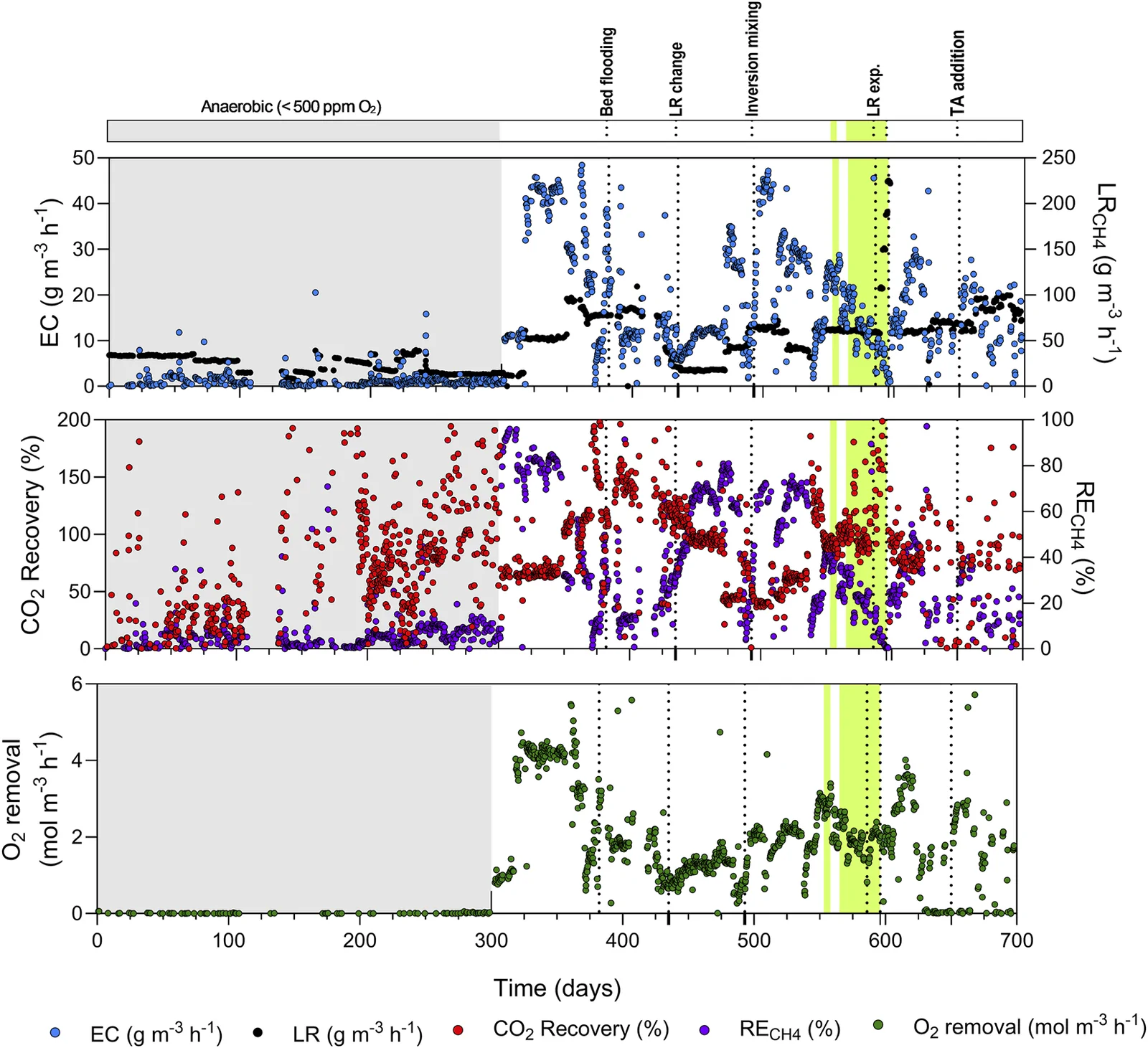
Establishment and long-term methane removal performance of a thermoacidophilic biotrickling filter (R1). Reactor 1 (R1, ‘Anaerobic’, grey shading) was initially packed with glass beads (Days 0–120) and operated without air but was found to contain <500 ppm O_2_ concentration, due to diffusion/leakage into the reactor. On day 300 the inlet O_2_ was increased to 4% and on day 437% to 5% (v/v). Periods of chemostat operation (D = 0.11–0.16 h^-1^) are shown (green shading). If not otherwise indicated the reactor sump was operated in batch mode. Vertical dashed lines indicate instances of bed flooding, bed mixing by inversion, loading rate (LR) changes, LR experiments (LR exp.) and thionine acetate (TA) addition. Reactor 2 (R2) was operated under ‘microaerophilic’ conditions (1.5%–3% O_2_, v/v), while Reactors three and 4 (R3, R4) were operated aerobically (8%–12% O_2_, v/v). Long-term performance of R2, R3 and R4 are provided in Supplementary Figure S3-5.

### 3.2 BTF reactors displayed variable performance during long-term operation

Throughout 700 days of continuous operation, all the BTF reactors displayed variable performance in response to experimental parameter changes (Tables 1; Supplementary Table S1). In general, the ‘microaerobic’ reactors (R1 and R2) displayed variable ECs, which were also reflected in the observed CO_2_ recovery and O_2_ removal values. For 33 days (starting on day 310), the EC of reactor R1 (Figure 2) was stable for the longest period; with an average EC of 41.9 (±2.6) g m^-3^ h^-1^ at a LR of 52.4 (±1.3) g m^−3^ h^−1^ after switching from low O_2_ < 0.1% conditions. In comparison R2’s EC was more unstable with the EC generally ranging from 10–30 g m^-3^ h^-1^ (Supplementary Figure S3). For the two aerobic reactors, the ECs generally ranged from ∼10–40 g m^-3^ h^-1^ (Supplementary Figures S4,5), but with occasionally periods of high EC after bed mixing in R4. R4’s EC was much more variable than R3. In R2, CO_2_ recovery was >100% for 154 out of 700 days. Similar periods of CO_2_ recovery in excess of 100% were observed in R3 and R4. This can occur when bacteria catabolise internal storage polymers (e.g., glycogen, PHA) accumulated during prior periods of unbalanced growth, i.e., O_2_ or nitrogen limitation and carbon excess (Carere et al., 2017; Pieja et al., 2011; Strange, 1968). *Methylacidiphilum* can store or degrade glycogen during unbalanced growth (Carere et al., 2019). In related work, glycogen degradation inhibitors limited *Methylacidiphilum* metabolism in the absence of methane (Gilbertson et al., 2025). Other possible contributing factors to CO_2_ recovery temporarily exceeding 100% included excreted metabolites or EPS degradation or heterotrophic scavenging. Changes in CO_2_ recovery were also magnified at low EC values, due to measurement uncertainties affecting their calculation (Supplementary Equation S3) and leading to recoveries temporarily exceeding 100% at the low EC conditions.

### 3.3 BTF microbial community structure and diversity

Following the initial enrichment period (43 days), reactor communities in R2-R4 (R1 not sampled) were dominated by *Methylacidiphilum* spp. (47–99% relative abundance) (Figure 3), a genus of aerobic thermoacidophilic methanotrophs (Phylum Verrucomicrobiota) known to grow between 40 °C–60 °C and pH 1–6 (Dunfield et al., 2007; Schmitz et al., 2021). In comparison, the geothermally heated soils used to inoculate each BTF reactor were dominated by non-methanotrophic thermoacidophilic taxa including *Acidithiobacillus* (80%), *Ferroplasma*, *Acidiplasma* and *Sulfobacillus* (Dopson et al., 2004; Golyshina et al., 2009; Panyushkina et al., 2019). Despite the low abundance (0.54%) of verrucomicrobial taxa in the soils used to inoculate each reactor, *Methylacidiphilum* spp. have previously been isolated throughout the Rotokawa geothermal region (Carere et al., 2017).

**FIGURE 3:**
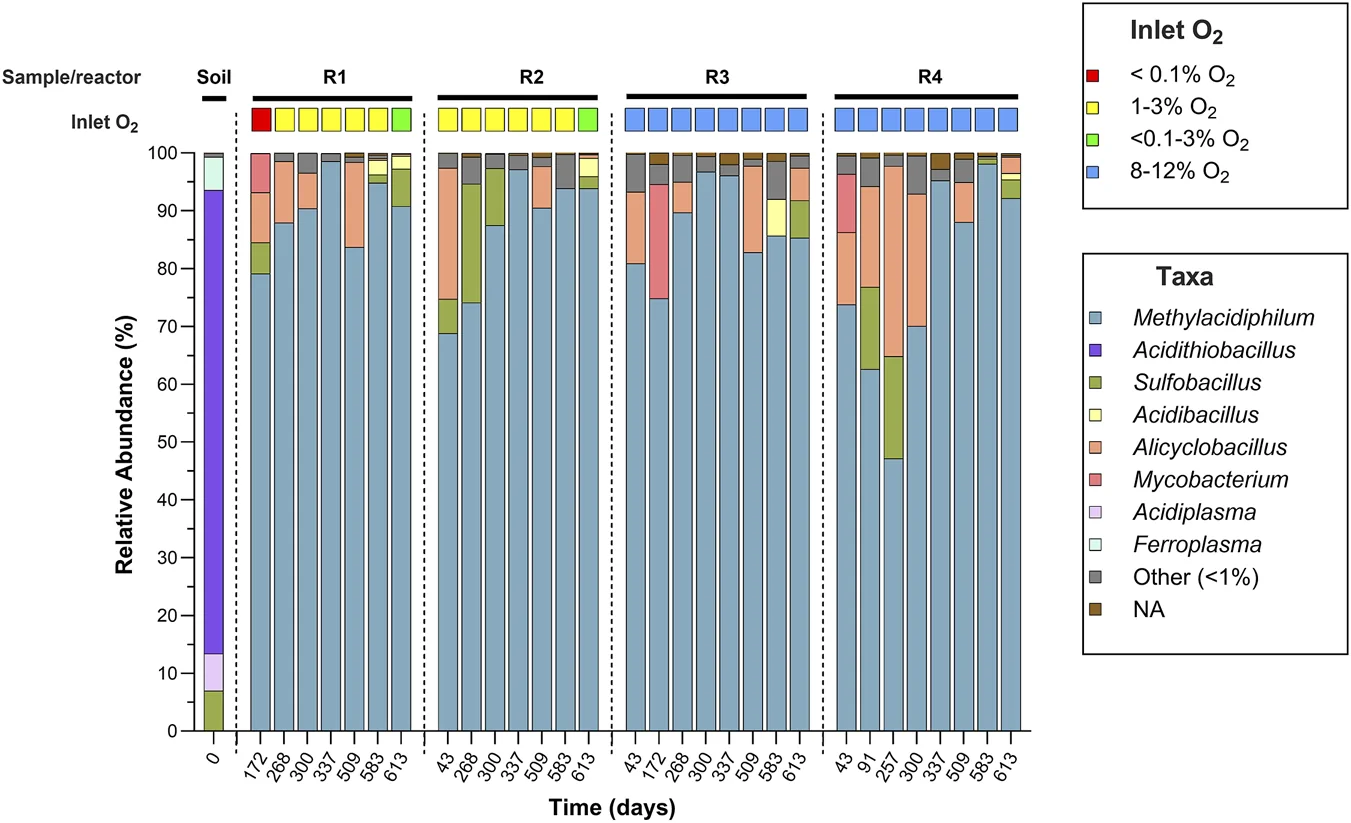
**–** Thermoacidophilic (45 °C, pH 2.2–2.5) methane-oxidising BTF reactor biofilm community composition (genus level) for reactors R1, R2, R3 and R4 over a >600-day operation period. The initial soil inoculum composition is also shown (Soil, day 0). The SILVA rRNA database v132 was used to assign taxonomic identity to amplicon sequences. ‘Other’ corresponds to genera at < 1% abundance. ‘NA’ represent taxa unclassified at the genus level. Inlet reactor O_2_ concentration (% v/v) is shown.

Microbial communities in biofilters and sequencing batch reactors vary temporally, spatially, and in response to changes to operating conditions (Cassarini et al., 2019; Henriet et al., 2017; Sercu et al., 2005). Nevertheless, reflecting the strong selective pressures of CH_4_ supply and thermoacidophilic operation, *Methylacidiphilum* remained the dominant genera (>47%) in all reactors over 2 years of continuous operation. The abundance of non-methanotrophic taxa, including *Alicyclobacillus*, *Sulfobacillus*, *Acidibacillus,* and *Mycobacterium*, fluctuated throughout operation (ranging from <1% to 32% relative abundance). *Alicyclobacillus* spp. are commonly identified in geothermal soils and are known to grow with *Methylacidiphilum* spp. (Kaparaju and Rintala, 2013). Likewise, *Sulfobacillus* are metabolically versatile thermoacidophiles (Panyushkina et al., 2019), commonly found in acidic and metal/sulfur-rich environments (e.g., volcanic solfatara vents) (Kondrat’eva et al., 2012). Both *Sulfobacillus* and *Alicyclobacillus* are well studied thermoacidophiles isolated from geothermal sites (Aulitto et al., 2021; Johnson, 2009; Panyushkina et al., 2019). Interestingly, in the first 6 months of operation, *Mycobacterium* spp. were detected at 5%–20% abundance in reactors R1, R3 and R4. *Mycobacterium* are aerobic bacteria not commonly associated with extremophilic conditions. Although thermophilic isolates are capable of growth up to 60 °C (pH 4.5), no *Mycobacterium* spp. are known to grow below pH 2.5 (Alarico et al., 2020; Gouzy et al., 2021).

As expected, diversity indices (Shannon Entropy, H' < 2.5; Simpson, D < 1) for the geothermal soil inoculum and BTF reactors indicated low species richness and evenness. Geothermal soils commonly display lower microbial diversity, compared to non-extreme soils (pH 7–8, 20–30 °C) (Sharp et al., 2014) due to their harsh physicochemical environment (e.g., pH < 4, 40 °C–100 °C). In comparison, non-extreme microbial communities within methane removing BTFs, are far more diverse (H′ 2.5–2.8) (Estrada et al., 2014). H_2_S removing BTFs have also displayed greater diversity (H′ 7.5–8), despite operating under mildly acidic conditions (pH 4) (Tu et al., 2016). Elucidating the influence of specific environmental factors (i.e., pH and temperature) on microbial diversity is difficult, due to the inherent influence from other variables such as soil type, chemical composition, and gases present (Picone et al., 2020).

Interestingly, following enrichment, the BTFs consistently displayed greater microbial diversity (H′ 1.49–1.74, D 0.66–0.74) than the geothermal soil inoculum (H′ 0.99, D 0.38). Previous reports of mesophilic biofilters have generally reported decreased diversity from the original inoculum following the application of a selection pressure (Cassarini et al., 2019). In these studies, large proportions of the community (>20%) were often unclassified (at the genus level) or low abundance (<1%) (Almenglo et al., 2016; Fan et al., 2021). Contrasting this, in the current study, unclassified/other genera comprised a relatively small fraction of the community (<5%) in both the soil and BTF. Thus, while it was possible soil DNA extractions underrepresented the initial microbial diversity (Frostegård et al., 1999; Högfors-Rönnholm et al., 2018; Xie et al., 2018), it seemed likely the thermoacidophilic conditions and availability of CH_4_ gas in BTF filters promoted the proliferation of low abundance (<1%) microorganisms from the soil.

### 3.4 Effect of methane loading rate on elimination capacity

To determine the maximum elimination capacity (EC_max_), the methane loading rate (LR) was increased stepwise between ∼57–350 g m^-3^ h^-1^ in ‘aerobic’ reactors R3 and R4 (10% inlet O_2_) and ‘microaerobic’ reactor R1 (2.5% inlet O_2_) by increasing the inlet CH_4_ concentration from ∼1.6% to 7%. During this period, the liquid sumps were run as chemostats to prevent liquid medium nutrient limitation (e.g., NH_4_^+^). Reactor R2 was not used for LR experiments due to constraints on available equipment. The ECs in R3 and R4 increased with increasing LR and the EC_max_ values in R3 and R4 were 30.7 (±2.4) and 18.6 (±3.2) g m^-3^ h^-1^, achieved at LRs of 155.1 and 236.3 g m^-3^ h^-1^, respectively (Figure 4). The EC of reactor R1 was less sensitive to LR changes up 100 g m^−3^ h^-1^, displaying an EC_max_ of 7.8 (±1.6) g m^-3^ h^-1^. These EC_max_ values were similar to non-extreme CH_4_ removing BTFs (Khabiri et al., 2022; Pryor et al., 2026), but lower than observed in a recent non-acidophilic thermophilic biofilters reporting a maximum EC of 75 g m^-3^ h^-1^ at 40 °C–60 °C (Gómez-Borraz et al., 2025) and 182 g m^-3^ h^-1^ at 50 °C (Avila-Nuñez et al., 2026).

**FIGURE 4:**
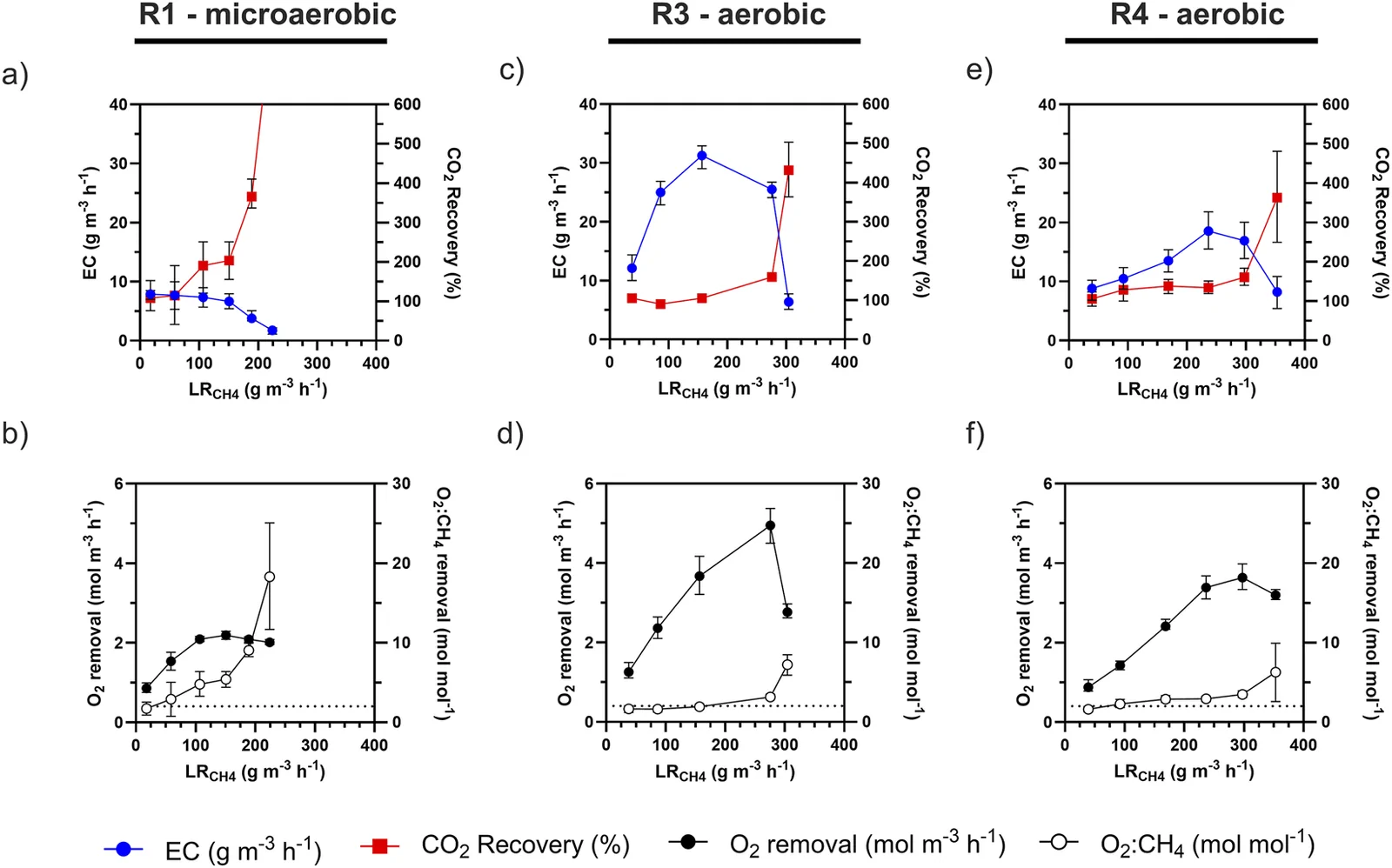
The influence of methane loading rate (LR_CH4_) on the elimination capacity (EC), CO_2_ recovery, O_2_ removal, and O_2_:CH_4_ removal ratio for ‘aerobic’ (10% inlet O_2_) BTF reactors R3 and R4 (right) and ‘microaerobic’ (2.5% inlet O_2_) reactor R1. All BTF reactors were maintained at 45 °C and continuously supplied with V4 medium (pH 2.2–2.5, D = 0.11 h^-1^) to prevent nutrient limitation (e.g., NH_4_^+^). The dashed horizontal line (bottom panels) indicates the theoretical stoichiometric O_2_ requirement for complete CH_4_ oxidation (2 mol_O2_ mol_CH4_^−1^). Average values are shown with error bars representing one standard deviation (n = 3–10 technical measurement replicates).

For all three reactors, the EC decreased at higher loading rates. This behaviour was unexpected, as ECs typically increase up to a critical LR, after which the EC remains constant with increasing LR except for cases of substrate inhibition (Devinny et al., 1998). A plateau in EC at high LR is the typical response in methane biofilters (Ferdowsi et al., 2016; Gómez-Cuervo et al., 2016; Khabiri et al., 2020). However, Gunasekera et al. (2018) did find that methane EC decreased when the LR exceeded 67 g m^-3^ h^-1^ and attributed it possibly to EPS hindering mass transfer. The decline in ECs at higher loading rates was possibly attributable to suboptimal O_2_:CH_4_ ratios constraining complete methane oxidation, with the possible additional contribution of toxic intermediate accumulation (e.g., formaldehyde, formate) under carbon-excess conditions (Chen et al., 2016; Chi et al., 2012; de Almeida et al., 2025), however further investigation is required.

During LR experiments, the observed CO_2_ recovery relative to methane consumption indicated the oxidation of non-CH_4_ compounds. For both reactors R3 and R4, CO_2_ recovery was >100% at LR > 100 g m^-1^ h^-1^ and rapidly increased to >380% at LR > 300 g m^-1^ h^-1^. Likewise, reactor R1 displayed >100% CO_2_ recovery at LR > 50 g m^-1^ h^-1^. Typically in methane BTFs, the CO_2_ recovery is near or less than 100% (Bordoloi and Gostomski, 2019; Gómez-Borraz et al., 2025; Khabiri et al., 2020), therefore some other compounds were likely being degraded at the higher loading rates, alongside the decreased methane degradation.

The rate of O_2_ consumption and the stoichiometry of O_2_ removal (O_2_:CH_4_, mol mol^-1^) was also consistent with a shift in microbial activity to a new carbon source (Figure 4). The stoichiometry of O_2_ removal during complete CH_4_ oxidation is theoretically 2 mol mol^-1^. The BTF reactors O_2_:CH_4_ removal stoichiometries exceeded two at LRs >100 g m^−3^ h^-1^, and the rate generally stayed high even as methane degradation decreased, indicating the occurrence of secondary aerobic processes. *Methylacidiphilum* sp. are known to accumulate intercellular glycogen under O_2_ and/or nitrogen limitation (Carere et al., 2017) and the oxidation of these reservoirs was consistent with the O_2_ removal and >100% CO_2_ recoveries observed. HPLC analysis of R1 and R4 liquid sump samples revealed the accumulation of formic acid up to 5.6 (±1.0) mM. It was possible that methane metabolism was inhibited by formic acid accumulation when the O_2_:CH_4_ feed ratio become less than sufficient but *Methylacidiphilum* was able to continue to metabolise glycogen. *Methylacidiphilum* is reported to be inhibited by formate exceeding 1 mM (Carere et al., 2021). R1 displayed this restricted methane degradation at a lower loading rate which is consistent as R1 was fed with 4% oxygen compared to 10% in R3 and R4. Inhibition at high methane concentrations and associated lower oxygen concentration is normally minor in non-thermoacidophilic methanotrophs even at concentrations exceeding the 6%–7% maximum methane concentration used in this work (Lee et al., 2011; Zhao et al., 2009).

Other abundant taxa identified in BTF reactors are capable of mixotrophic (e.g., *Acidithiobacillus* spp. (Aston et al., 2011)) or heterotrophic (e.g., *Sulfobacillus* spp., (Tsaplina et al., 2008); *Acidibacillus* spp., (Aston et al., 2011); *Alicyclobacillus* spp. (Yahya et al., 2008)) growth. Therefore, it is also plausible that the oxidation of accumulated excreted metabolites was contributing to the inflated CO_2_ recoveries and O_2_:CH_4_ removal stoichiometries at faster methane loading rates, but metatranscriptomic and/or metabolomic analysis would be required to confirm this hypothesis.

### 3.5 Iron dissolution supported Fe-dependent taxa

Within the first 65 days of operation, the metal packing supports used in all reactors rusted and thus were replaced with plastic counterparts. Concomitant to rusting, orange-coloured precipitants on the pumice rock packing and proximal to the metal supports were observed. Although rusted components were removed, some stainless-steel parts (e.g., piping) continued to be used throughout operation.

Periodic MP-AES analysis of sump fluids revealed elevated soluble Fe concentrations (up to 160 ppm compared to 2 ppm in V4 medium) in all reactors, that were consistent with the observed corrosion of metal tubes and reactor supports. While 316 stainless steel componentry was used in this study, material imperfections and exposed Fe from unsealed threads/cuts may have caused abiotic or bacterial metal dissolution. Crevice and pitting corrosion due to microbially induced corrosion of stainless steel is well-known, especially on metal interfaces between anoxic and oxic zones (Javed et al., 2022). Moreover, microaerobic conditions can create low redox potentials that reduce passivation layers on mineral surfaces, which can promote mineral dissolution by acidophiles (Donati et al., 2016). In all reactors, *Sulfobacillus* and *Alicyclobacillus* were the second most abundant genera. Both are well known iron oxidizers/reducers (Jiang et al., 2008; Panyushkina et al., 2019), with some species capable of mixotrophic growth on Fe^2+^ with organic carbon (Karavaiko et al., 2001; Ñancucheo and Johnson, 2010) and/or Fe-mineral dissolution (Malik and Hedrich, 2022). Considering the potential for *Methylacidiphilum* spp. to excrete organic acids under oxygen or nitrogen limitation (Khadem et al., 2012), it is tempting to speculate syntrophic interactions in which chemolithoautotrophic species contributed to the observed metal corrosion. Still, metal dissolution driven by methane oxidation in mixed cultures under thermoacidophilic conditions has never been reported. The liberation of Fe^2+^/Fe^3+^ is likely to have both abiotic and biotic origins in the reactor. At pH < 2.5 these processes were likely accelerated by sulfate-reducing organisms (e.g., *Ferroplasma* spp.) (Bridge and Johnson, 1998).

### 3.6 Periodic behaviour

Reactor three experienced the most persistent periodic behaviour, aligning with medium changes every 7–14 days (Supplementary Figure S8). The EC, O_2_ removal and CO_2_ recoveries followed similar patterns, increasing rapidly (1–2 days) after changing medium then gradually decreasing after reaching a maximum EC between 40–55 g m^-3^ hr^-1^. The peak ECs in each cycle were greater than the EC_max_ from the LR study of 30.7 ± 2.4 g m^-3^ hr^-1^. Increases in EC after inversion mixing of Reactors 2–4 suggested mass transfer limitation, as the trend was observed across the microaerobic and aerobic columns (Supplementary Figures S9,10). Intermittent bed flooding between days 158–230 in Reactors 2–4 led to instantaneous drops in EC by 80%–90%, also supporting this hypothesis (Supplementary Figures S11, 12). Previous studies on mesophilic methane BTFs have demonstrated that mass transfer from the liquid to the biofilm is the rate limiting step with polyurethane foam packings (Estrada et al., 2014).

It was assumed that the liquid recycling in BTFs was effectively covering the entire bed and providing liquid nutrients to the biofilm surface to support methane degradation. Improper liquid distribution can lead to channelling, stagnant portions of the bed with poor liquid nutrient renewal and reduced active surface area (Trejo-Aguilar et al., 2005). Tracer compounds can be used to determine the effective wetted surface area and void fraction in BTFs (Sharvelle et al., 2008). Addition of the redox dye thionine acetate (TA), in related work in these columns (Gilbertson et al., 2025), revealed considerable liquid channelling in reactors R1 and R2 (Supplementary Figure S12). Moreover, 1–2 cm void spaces developed in the filter bed over time, specifically after bed flooding (Supplementary Figure S13). These results suggest that periodic behaviour observed in all reactors over 2 years was likely due to poor gas and liquid distribution, limiting the effective active biofilm in the BTFs. This is a recognised problem in trickle-bed reactors (Shah, 1979).

## 4 Conclusion

In this study we demonstrated the successful enrichment of thermoacidophilic methane removing cultures within a BTF under varying oxygen concentrations. The maximal EC under non−limiting aerobic conditions was 18.6 (±3.2) and 30.7 (±2.4) g m^−3^ h^−1^ at CH_4_ loading rates of 236.3 and 155.1 g m^−3^ hr^−1^, respectively. These EC values decreased at higher LR rates possibly due to inhibition from formic acid accumulation. The greatest sustained EC, 41.9 (±2.6) g m^−3^ h^−1^ at a LR of 52.4 (±1.3) g m^−3^ h^−1^, was observed under microaerobic (1%–3% O_2_, R1) conditions, over a 33-day period after switching from low O_2_ < 0.1% conditions. Unstable ECs were observed in all reactors throughout operation, with reactors periodically increasing EC in response to medium replenishment or biofilm removal. These non-steady state ECs were attributed to shifts in metabolism from methane oxidation to internal storage polymer accumulation in response to excessive biofilm accumulation and mass transfer limitations deep in the biofilm but would require confirmation by metatranscriptomic and/or metabolomic analysis. The microbial communities in all reactors were dominated (47%–99% relative abundance) by known thermoacidophilic methanotrophs (*Methylacidiphilum* spp.), with the intermittent presence of other acidophilic genera (e.g., *Alicyclobacillus, Sulfobacillus,* and *Acidibacillus*) also observed. These findings highlight the potential of thermoacidophilic BTFs for mitigating methane emissions from acidic and high-temperature sources such as landfills and geothermal soils.

Future work with this system should explore packing and reactor modifications that account for the excess biomass production. The EC values were similar to rates reported for many mesophilic methane-removing systems, but the channelling results indicated that the columns were likely operated at sub-optimal conditions, indicating the potential for achieving higher EC values. However, the thermoacidophilic community may not be suitable for high methane loading rates due to potential formic acid accumulation and inhibition in the recycled medium in the BTF. Excess biomass production may be a larger issue compared to mesophilic methanotroph BTFs due to potential pressure drop problems. However, they may have applications in systems with lower methane concentrations such as emissions associated with mining or cattle barn operations, where continued growth may enhance the elimination capacity.

## Data Availability

The raw data supporting the conclusions of this article will be made available by the authors, without undue reservation.
