# Supplementary material for: Long-term performance of thermoacidophilic methanotrophic biotrickling filters
Source: Front Bioeng Biotechnol. 2026 Oct 29;14:1954174. doi: 10.3389/fbioe.2026.1954174 (PMC13755692; doi:10.3389/fbioe.2026.1954174)
Supplement: Supplementary file 1 [file Supplementaryfile1.docx]

Relevant biofilter equations

The elimination capacity (EC) of CH_4_, CH_4_ removal efficiency (RE), CH_4_ loading rate (LR), volume of empty filter bed *V_f_* (m^3^), volumetric gas flow rate *Q* (m^3^ min^-1^), inlet concentration *C_i_* (% v/v), outlet gas concentration *C_o_* (% v/v), universal gas constant R (8.314 m^3^ Pa K^-1^ mol^-1^), temperature *T* (K), inlet gas pressure *P* (Pa), and CH_4_ molecular weight *MW* (g mol^-1^), empty bed residence time (EBRT). For EC in mole units the *MW* is left out of Equation S1.

**Table S1 -** Reactors R1, R2, R3 and R4 gas flow rates and inlet CH_4_ & O_2_ concentrations and bed packing materials between days 0 and 700. Uncertainties represent one standard deviation (n > 25).

| **Reactor** | **Days** | **Inlet CH_4_ ^a^**  **(% v/v)** | **Inlet O_2_ ^a^**  **(% v/v)** | **Gas flow rate ^a^**  **(mL min^-1^)** | **CH_4_ Loading Rate ^a^**  **(g_CH4_ m^-3^ h^-1^)** | **Operational notes** |
| --- | --- | --- | --- | --- | --- | --- |
| R1 | 0 – 120 | 4.0 (± 0.12) | 0.05 ^†^ | 10.8 (± 1.1) | Min: 15.79 (± 1.47)  Max: 34.04 (± 0.26) | Glass bead packing ^‡^ |
|  | 0 – 299 | Min: 1.75 (± 0.30),  Max: 4.09 (± 0.15) | 0.05 ^†^ | Min: 5.0 (± 0.1),  Max: 24.0 (± 7.2) | Min: 15.79 (± 1.47)  Max: 34.04 (± 0.26) | Anaerobic enrichment ^†^ |
|  | 30 – 55 ^b^ | 4.00 (± 0.07) | 0.05 ^†^ | 11.7 (± 0.1) | 33.48 (± 0.48) |  |
|  | 75 – 100 ^b^ | 4.14 (± 0.09) | 0.05 ^†^ | 9.4 (± 0.1) | 27.25 (± 2.95) |  |
|  | 300 – 700 ^a^ | 1.83 (± 0.83) | Min: 2.57 (± 0.62),  Max: 4.78 (± 0.21) | Min: 12.5 (± 0.5),  Max: 59.3 (± 3.5) | Min: 10.5(± 0.53)  Max: 225.2 (± 1.08) | Microaerobic operation |
|  | 430 – 470 | 0.58 (± 0.07) | 4.03 (± 1.03) | 28.5 (± 5.23) | 18.64 (± 2.30) | Leakage * |
|  | 586 – 598 | Min: 1.79 (± 0.02),  Max: 7.01 (± 0.03) | 2.72 (± 0.07) | 43.5 (± 0.01) | Min: 57.5 (± 0.55)  Max: 225.2 (± 1.08) | LR experiments |
|  | 650 – 700 | 2.46 (± 0.37) | 2.66 (± 0.68) | 41.9 (± 7.1) | 80.13 (± 12.07) | Thionine acetate added |
| R2 | 0 – 120 | 3.71 (± 0.21) | 1.82 (± 0.53) | Min: 8.4 (± 0.1),  Max: 20.0 (± 0.2) | Min: 22.56 (± 0.27)  Max: 55.75 (± 1.16) | Microaerobic enrichment,  Glass bead packing^‡^ |
|  | 0 – 700 | 2.10 (± 0.94) | 2.56 (± 0.93) | Min: 8.4 (± 0.1),  Max: 87.6 (± 2.9) | Min: 22.15 (± 0.27)  Max: 238.9 (± 13.08) | Microaerobic operation |
|  | 30 – 55 ^b^ | 3.56 (± 0.14) | 2.07 (± 0.56) | 13.5 (± 0.1) | 35.35 (± 1.51) |  |
|  | 75 – 100 ^b^ | 3.80 (± 0.08) | 2.00 (± 0.59) | 8.4 (± 0.1) | 24.91 (± 8.02) |  |
|  | 430 – 470 | 0.56 (± 0.13) | 3.14 (± 0.26) | 57.9 (± 0.9) | 22.87 (± 0.79) | Leakage * |
|  | 650 – 700 | 2.05 (± 0.15) | 3.05 (± 0.30) | 52.7 (± 2.2) | 80.57 (± 6.68) | Thionine acetate added |
| R3 | 0 – 120 | 2.33 (± 0.21) | 10.27 (± 1.09) | Min: 11.2 (± 0.1),  Max: 19.6 (± 1.2) | Min: 18.5 (± 1.12)  Max: 83.6 (± 1.80) | Aerobic enrichment |
|  | 0 – 700 | 2.33 (± 0.21) | 9.07 (± 1.79) | Min: 11.3 (± 0.7),  Max: 91.7 (± 3.7) | Min: 18.23 (± 1.12)  Max: 304.5 (± 1.27) | Aerobic operation |
|  | 30 – 55 ^b^ | 2.52 (± 0.17) | 10.57 (± 0.32) | 20.2 (± 0.7) | 31.80 (± 1.11) |  |
|  | 75 – 100 ^b^ | 2.43 (± 0.12) | 10.13 (± 0.61) | 11.3 (± 0.1) | 23.09 (± 6.65) |  |
|  | 430 – 470 | 0.69 (± 0.02) | 9.98 (± 0.16) | 53.7 (± 5.7) | 35.64 (± 2.09) |  |
|  | 586 – 598 | Min: 1.59 (± 0.02),  Max: 6.21 (± 0.03) | 9.94 (± 0.21) | 67.5 (± 0.01) | Min: 76.3 (± 0.59),  Max: 304.5 (± 1.27) | LR experiments |
| R4 | 0 – 120 | 2.40 (± 0.29) | 9.72 (± 0.79) | Min: 12.8 (± 0.1),  Max: 65.4 (± 1.2) | Min: 16.2 (± 0.35)  Max: 85.2 (± 1.02) | Aerobic enrichment |
|  | 0 – 700 | 1.80 (± 0.85) | 8.59 (± 3.22) | Min: 12.4 (± 0.8),  Max: 96.1 (± 4.2) | Min: 20.6 (± 0.82)  Max: 353.2 (± 1.61) | Aerobic operation |
|  | 30 – 55 ^b^ | 2.28 (± 0.05) | 10.14 (± 0.27) | 20.1 (± 0.3) | 32.6 (± 1.04) |  |
|  | 75 – 100 ^b^ | 2.42 (± 0.32) | 9.94 (± 0.83) | 14.3 (± 3.3) | 24.7 (± 5.68) |  |
|  | 430 – 470 | 0.65 (± 0.03) | 9.00 (± 0.32) | 56.3 (± 4.4) | 38.3 (± 2.96) | Leakage * |
|  | 586 – 598 | Min: 1.72 (± 0.02),  Max: 6.62 (± 0.03) | 8.91 (± 0.21) | 76.4 (± 7.4) | Min: 91.7 (± 0.82)  Max: 353.2 (± 1.61) | LR experiments |

^‡^ Scoria packing was used from days 120 – 700

^†^ Periods of anaerobic operation contained < 500 ppm O_2_ due to diffusion

^a^ Where indicated, average minimum (Min) and maximum (Max) range of operational parameter setpoints are provided.

^b^ Start-up operational period illustrated in Figure 1

*Operational issue due to leakage in calibration gas supply.

**Figure S1 -** P&ID of laboratory-scale thermoacidophilic BTF reactor system. Only one reactor (of 4 total) is shown.

Gas Flow

Liquid Flow

**Figure S2 -** Photograph of a fully assembled and inoculated BTF reactor (left), polytetrafluoroethylene (PTFE) plates perforated with 1 mm holes to support the packing material (top right) and modified round bottom flask liquid sumps containing circulating V4 medium (bottom right).

**Figure S3** – Establishment and long-term methane removal performance of a thermoacidophilic biotrickling filter (R2). Reactor 2 was operated as a ‘microaerobic’ BTF with an inlet O2 concentration of 1.5-3.0 % (v/v). Throughout the entire period of operation, the reactor sump was operated in batch mode, with perodic replacement of V4 liquid medium. Vertical dashed lines indicate instances of bed flooding, bed mixing by inversion, loading rate (LR) changes, LR experiments (LR exp.) and thionine acetate (TA) addition.

**Figure S4 –** Establishment and long-term methane removal performance of a thermoacidophilic biotrickling filter (R3). Reactor 3 was operated as an ‘aerobic’ BTF with an inlet O2 concentration of 6.0-10.0 % (v/v). Periods of chemostat operation (D = 0.11 – 0.16 h-1) are shown (green shading). If not otherwsie indicated the reactor sump was operated in batch mode. Vertical dashed lines indicate instances of bed flooding, bed mixing by inversion, loading rate (LR) changes, LR experiments (LR exp.) and thionine acetate (TA) addition.

**Figure S5** – Establishment and long-term methane removal performance of a thermoacidophilic biotrickling filter (R4). Reactor 4 was operated as an ‘aerobic’ BTF with an inlet O_2_ concentration of 6.0-10.0 % (v/v). Periods of chemostat operation (D = 0.11 – 0.16 h^-1^) are shown (green shading). If not otherwsie indicated the reactor sump was operated in batch mode. Vertical dashed lines indicate instances of bed flooding, bed mixing by inversion, loading rate (LR) changes, LR experiments (LR exp.) and thionine acetate (TA) addition.

**Figure S6.** Biofilm development in thermoacidophilic (45 °C, pH 2.5) BTF Reactor 2 under microaerobic (2 % inlet O_2_, v/v) conditions during the first 180 days of operation using pumice stone as the packing material.

**Figure S7.** Biofilm development in thermoacidophilic (45 °C, pH 2.5) BTF Reactor 3 under aerobic (8-12% inlet O_2_, v/v) conditions during the first 180 days of operation using pumice stone as the packing material.

**Figure S8.** Periodic behaviour in EC, CO_2_ recovery and O_2_ removal in Reactor 3. Black triangles indicated V4 medium replacement and red triangles are “Rain Sticking” events to dislodge excess biomass.

**Figure S9.** Periodic behaviour in EC, CO_2_ recovery and O_2_ removal in Reactor 2. Black triangles indicated V4 medium replacement and red triangles are “Rain Sticking” events to dislodge excess biomass.

**Figure S10.** Periodic behaviour in EC, CO_2_ recovery and O_2_ removal in Reactor 4. Black triangles indicated V4 medium replacement and red triangles are inversion mixing events to dislodge excess biomass.

**Figure S11**. The performance of Reactor 2 during bed flooding (yellow triangle). Vertical black dashed lines indicate replenishment of V4 medium and inlet CH4 change (blue triangle).

**Figure S12.** Bed flooding (yellow) in aerobic Reactors 3 (top) and 4 (bottom) during chemostat operation day 188-230. Black triangles indicated V4 medium replacement and red triangles are inversion mixing events to dislodge excess biomass.

**Figure S12.** Microaerobic reactors 1 (left) and 2 (right) before and after addition of 500mM thionine acetate with 2-3% (v/v) inlet O2. Large portions of the bed unstained by thionine acetate (red circles).

**Figure S13.** Large voids in reactors before and after bed flooding in Reactor 2.
